# Clinical Differences between Early- and Late-Onset Asthma: A Population-Based Cross-Sectional Study

**DOI:** 10.1155/2021/8886520

**Published:** 2021-01-12

**Authors:** Jiaming Li, Ling Ye, Jun She, Yuanlin Song

**Affiliations:** Department of Pulmonary Medicine, Zhongshan Hospital, Fudan University, Shanghai, China

## Abstract

**Background:**

Limited information exists about the nature of late-onset asthma (LOA) without medication intervention when compared to early-onset asthma (EOA). Our goal was to understand how EOA and LOA affect clinical and pathophysiological features.

**Methods:**

A population-based cross-sectional study was carried out in Zhongshan Hospital (Shanghai, China). EOA and LOA were based on age of diagnosis (before and after age 40 years, respectively). Clinical variables were collected with an emphasis on allergic features, analyzed, related, and compared using one-way ANOVA or Kruskal-Wallis test. Correlations between blood basophils and clinical data were evaluated by Spearman's rank test. Statistical analyses were conducted with SPSS v24.0.

**Results:**

Of a total of 12,760 adults with cough, sputum, or chest tightness, 90 subjects with EOA (mean age ± standard deviation (SD):28.73 ± 5.89), 111 with LOA (mean age ± SD: 60.25 ± 9.85), and 106 with chronic obstructive pulmonary disease (COPD) (mean age ± SD: 61.58 ± 10.95) were selected. FEV_1_/FVC (%), FEV_1_% predicted, and FVC% predicted were all significantly lower in LOA compared to EOA (*p* < 0.01). The values of post-bronchodilator FEV_1_ in bronchodilator reversibility testing were higher in the LOA and EOA groups compared to subjects with COPD (*p* < 0.01). Among allergic features, mite sensitization was most common in EOA patients, followed by LOA and COPD, whereas mold sensitization was more prevalent in LOA than EOA. Moreover, blood eosinophils were a typical feature of asthma in both EOA and LOA compared to COPD and controls (*p* < 0.01), and there were no differences in blood neutrophils in LOA compared to controls. Interestingly, blood basophils were increased in both EOA (*p* < 0.01) and LOA (*p* < 0.05) compared to COPD and controls. This variable correlated with eosinophils in EOA (*r* = 0.549, *p*=0.002) but not in LOA.

**Conclusion:**

LOA is a distinct clinical entity from EOA. In LOA, atopy was less frequent and spirometry values were lower when compared to EOA. In EOA, blood basophils and eosinophils were significantly correlated owing to pathophysiological differences between the two forms of the disease.

## 1. Introduction

Asthma is one of the major global health problems and contributes substantially to the worldwide burden of disease [1, 2]. Prevalence of adult asthma ranges from 2.8% to 15.7% in young adults and up to 10% in older adults aged over 65 years. [3, 4] Onset is typically thought to occur during childhood or young adulthood, but 30–40% of the adults with asthma had their initial presentation after the age of 40 [5]. Up to 52% of asthmatic patients had their “first asthma attack” after 40 years of age [6]. Asthma that starts in middle or older age (late-onset asthma, LOA) is frequently undiagnosed; it is believed to be “normal” in old age due to lack of fitness and reduced activity. This may lead to delayed treatment [7]. About 20% of subjects with LOA previously had received a diagnosis of chronic obstructive pulmonary disease (COPD) [8].

Due to aging-related changes in respiratory pathology and immunity, LOA is frequently not diagnosed or is misdiagnosed [9]. Unlike early-onset asthma (EOA), atopic changes are unlikely in LOA, but the types of antigen sensitization exposures in LOA and EOA are unclear [10]. There is also a lack of characterization of differences in inflammation between LOA and EOA. While eosinophilic inflammation is a typical feature of asthma [11], in LOA, eosinophils may have decreased effector functions, but evidence on their role in airway hyper-responsiveness (AHR) is conflicting [12].

Most LOA patients enrolled in studies had been prescribed inhaled corticosteroids (ICS), oral corticosteroids (OCS), or leukotriene receptor blockers, which mask natural disease features and hamper research into the underlying mechanisms [13–15]. It is difficult to recruit patients with asthma at their initial presentation prior to pharmacologic interventions. Although LOA is associated with distinct phenotypic characteristics and immunologic levels, the factors associated with the development of LOA are unclear. In this study, we sought to better understand the factors associated with EOA and LOA, and their clinical and pathophysiological features in Chinese patients.

## 2. Materials and Methods

### 2.1. Study Setting and Design

A single-center, population-based, cross-sectional study was carried out in Zhongshan Hospital, Fudan University, Shanghai, China. This 2430-bed tertiary hospital is used by 10 million people in Shanghai and 150 million people in the surrounding Yangtze River Delta. More than 260,000 outpatients are referred to the Department of Pulmonary Medicine annually. All subjects signed informed consent forms and the study was approved by the Institutional Review Board, Human Subjects Research Protection Program Office (B2018-010R) at Zhongshan Hospital, Fudan University.

### 2.2. Study Subjects

**Figure 1:**
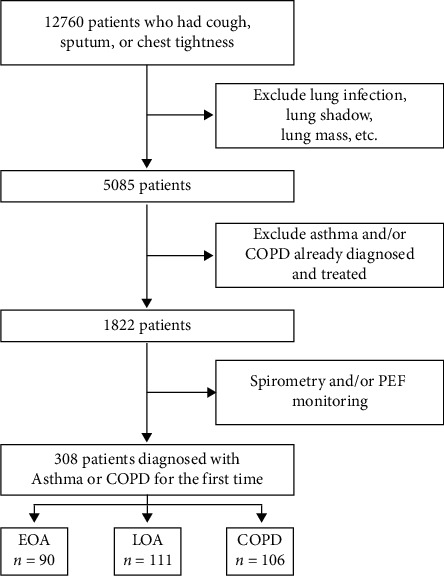
Study profile-The number of patients enrolled and analyzed in the study.

Subjects were screened from the Screening and Monitoring for Obstructive Lung Disease project from February 2018 to October 2019. A total of 12,760 patients with cough, sputum, or chest tightness were referred to the Department of Pulmonary Medicine. We excluded 5085 patients with lung infection, lung shadow at chest xRay, or lung mass, and 3260 patients with previous diagnosis of asthma and/or COPD or already on medication with drugs for chronic obstructive airway disease. The remaining 1822 patients underwent pulmonary function and/or bronchodilator reversibility testing. If the spirometry results did not support the suspicion of asthma, the patients were subjected to peak expiratory flow (PEF) monitoring (Circassia Medical Device Co., Ltd., Morrisville, NC, USA). Finally, 308 patients were diagnosed with asthma or COPD at the initial presentation by the pulmonologist or the attending physician according to the Global Initiative for Asthma (GINA) and the Global Initiative for Chronic Obstructive Lung Disease (GOLD) criteria [1, 16]. The study flow chart is given in Figure 1.

Before performing spirometry examination and collecting blood samples, patients under medication for other indications were asked to stop the related medication for at least 7–10 days (e.g., cough syrup, Chinese traditional medicine, and bronchodilator). Briefly, adults with respiratory symptoms, as indicated by an increase in forced expiratory volume in 1 second (FEV_1_) of >12% and >200 mL from baseline, 10–15 minutes after 200–400 mcg salbutamol (albuterol) or a change in PEF of at least 20% from baseline after 4 weeks of anti-inflammatory treatment, were considered as positive for diagnosis of asthma [1, 17]. A case was defined as EOA or LOA if the subject was diagnosed at age <39 or ≥40, respectively, as previously recommended [15, 18].

COPD was defined as any incidence of dyspnoea, chronic cough, or sputum production, with a history of recurrent lower respiratory tract infections and/or a history of exposure to risk factors for the disease and post-bronchodilator FEV_1_/forced vital capacity (FVC) < 0.7 with chronic respiratory symptoms [16, 19]. All patients with lung infection, lung shadow or mass, previous asthma and/or COPD diagnosis, and age <18 years were excluded. Healthy controls who reported no history of smoking or respiratory disease were recruited for the study.

### 2.3. Data Collection

Clinical information of all patients was carefully collected, including age, gender, weight, height, body mass index (BMI), smoking history (smoking ≥ 1 cigarette per day for 1 year), family history, related disease history, and asthma severity. Subjects with asthma were classified as mild, well controlled with step 1 and 2 treatments (e.g., monotherapy ICS or other single agent), moderate, well controlled with step 3 treatment (e.g., ICS plus one additional controller medication), or severe with step 4 or step 5 treatment (e.g., ICS plus ≥2 additional asthma control medications) based on the GINA criteria and National Institutes of Health guidelines [1, 20]. According to the Global Initiative for Chronic Obstructive Lung Disease–(GOLD) Classification of airflow limitation severity in COPD (based on post-bronchodilator FEV1), patients with FEV1/FVC < 0.70 were divided into mild (GOLD1), FEV_1_ ≥ 80% pred; moderate (GOLD2), 50% ≤ FEV_1_ < 80% pred; severe (GOLD3), 30% ≤ FEV_1_ < 50% pred; and very severe (GOLD4), FEV_1_ < 30% pred, based on post-bronchodilator FEV_1_values in the GOLD guidelines [16, 21].

All subjects underwent allergy testing for specific immunoglobulin E (sIgE) in serum (Uranus AE 65, China), and blood cell counts were performed [22]. Pulmonary function tests (Jaeger, Master Screen PFT, Germany) included FEV_1_% pred, FEV_1_ (L), FVC% pred, FVC (L), FEV_1_/FVC (%), and bronchodilator reversibility testing in accordance with the American Thoracic Society guideline [23]. Fractional exhaled nitric oxide (FeNO) was also measured (NIOX VERO, Circassia Medical Device Co., Ltd.).

### 2.4. Statistical Analysis

Clinical characteristics are reported as means ± standard deviation or percentages. Continuous variables were analyzed by one-way analyses of variance, and categorical variables were assessed with Kruskal-Wallis tests [19]. Correlations between the percentage of blood basophils with gender, BMI, smoking history, family history, rhinitis, white cell counts, the percentage and absolute values of blood eosinophils, the absolute value of blood basophils, the percentage and absolute values of blood neutrophils, FEV_1_% pred, FEV_1_ (L), FVC% pred, FVC (L), FEV_1_/FVC (%), FeNO, allergens, and sIgE were evaluated by Spearman's rank tests [11]. All tests were two-sided, and *p* values < 0.05 were considered statistically significant. Statistical analyses were conducted with SPSS for Windows, version 24.0 (IBM Corp., Armonk, NY, USA).

## 3. Results

### 3.1. Patient Characteristics

After enrollment and screening, a total of 308 patients were diagnosed with asthma or COPD at their initial presentation (Figure 1). Based on the age at initial presentation, a total of 90 patients were diagnosed with EOA (mean age 28.73 ± 5.89 years, range 18–39) and 111 patients with LOA (mean age 60.25 ± 9.85 years, range 41–80). Further 106 patients were diagnosed with COPD (mean age 61.58 ± 10.95 years, range 38–84). In the EOA and LOA groups, the gender distribution was roughly 60% male and 40% female, compared to approximately 70% and 30%, respectively, in the COPD group. The patients' clinical characteristics are listed in Table 1.

There were no differences in BMI among the groups. However, the subgroup of female patients with LOA had a higher BMI compared to female patients in the EOA group (*p* < 0.05). Subjects with LOA were less likely to report a diagnosis of allergic rhinitis compared to those with EOA (52.7% vs. 80%), and COPD patients had the highest rate of smoking history (62.3%) among the three groups. With regard to asthma severity, LOA patients were more likely to be categorized as moderate (52.3% vs. 46.7%%) or severe (25.2% vs. 14.4%) compared to those with EOA. In the COPD group, most of the patients belonged to GOLD1class (39.6%) compared to the other subgroups (29.2%, 24.5, 6.6% for GOLD 2, 3, and 4, respectively).

### 3.2. Lung Function Tests and Allergic Features

**Figure 2:**
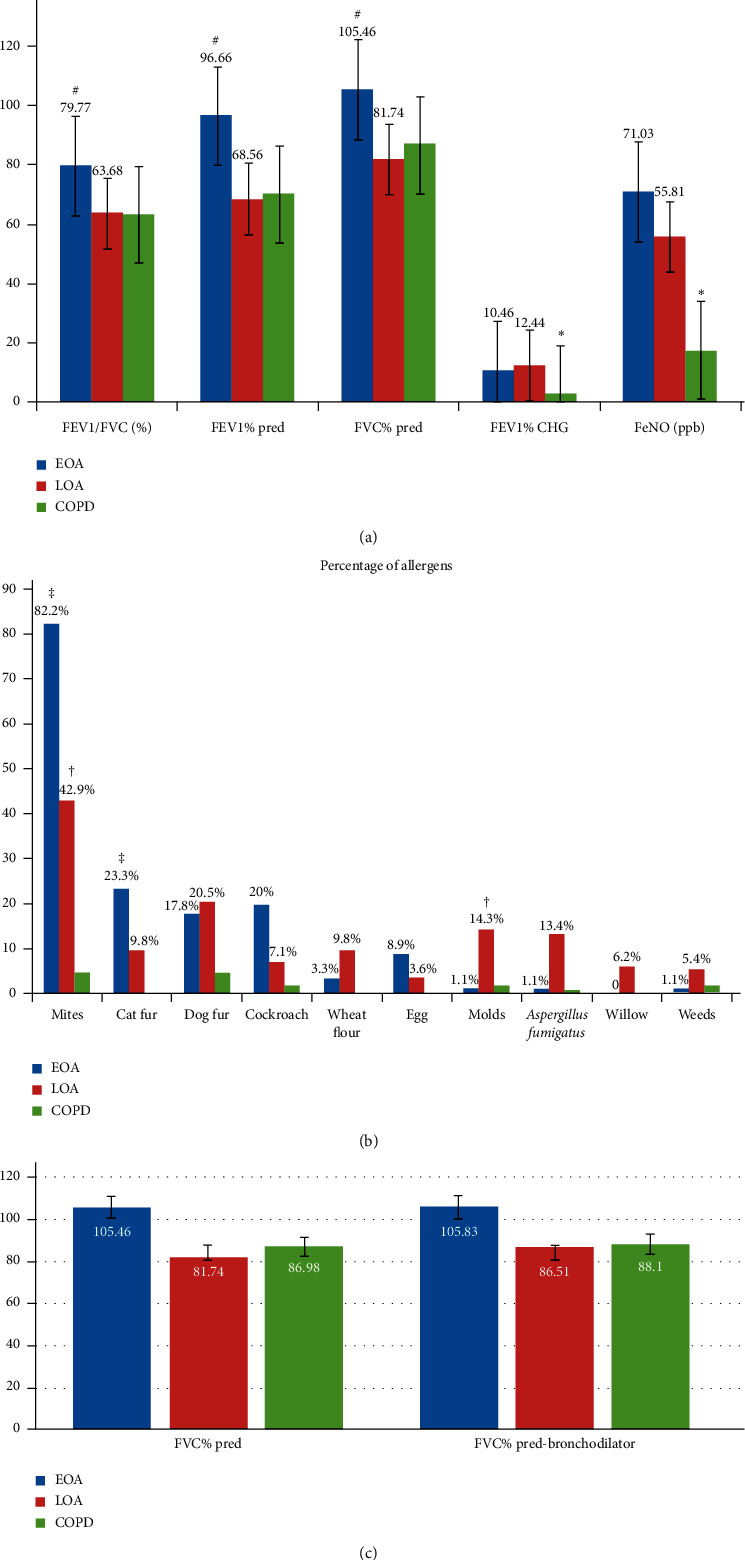
Lung function test and allergic features. (a) Spirometry results. ^#^*p* < 0.01 EOA vs. LOA; ^∗^*p* < 0.01 EOA and LOA vs. COPD. (b) The percentage of allergens. ^†^*p* < 0.05 LOA vs. EOA and COPD; ^‡^*p* < 0.01 EOA vs. COPD. (c) Reversibility for FVC.

The lung function test and allergic features were depicted in Figure 2(a)–2(c). FEV_1_/FVC (%), FEV_1_% pred, and FVC% pred were all significantly lower in LOA compared to EOA (*p* < 0.01). The values of post-bronchodilator FEV_1_ in bronchodilator reversibility testing were higher in the LOA and EOA groups compared to subjects with COPD (*p* < 0.01). Moreover, although FeNO was lower in LOA compared to EOA, both groups showed significantly higher FeNO compared to the COPD group (*p* < 0.01). Respiratory physiology information is shown in Figure 2(a) and Supplementary Table S1.

There was a marked difference in allergic sensitization between the LOA and EOA groups (Figure 2(b), Supplementary Table S1). Sensitization to mites was most common in EOA patients (*n* = 74, 82.2%, *p* < 0.01), followed by LOA (*n* = 48, 42.9%, *p* < 0.05) and COPD (*n* = 5, 4.7%). However, more subjects with LOA showed sensitization to mold compared to EOA (14.3% vs. 1.1%, *p* < 0.05). Furthermore, the LOA group exhibited more sensitivity to *Aspergillus fumigatus* than the EOA group (13.4% vs. 1.1%, *p*=0.053).

### 3.3. Inflammatory Blood Cell Counts

As shown in Table 2, the levels of blood eosinophils (absolute values and percentages) were increased in both LOA and EOA compared to COPD and healthy controls (*p* < 0.01). Interestingly, blood basophils (absolute values and percentages) were also increased in both LOA and EOA. Blood basophil levels were the highest in EOA patients (*p* < 0.01), followed by LOA (*p* < 0.05), compared to COPD and healthy controls. There were no differences in blood neutrophils (absolute value and percentage) among the LOA, EOA, COPD, and healthy control groups.

### 3.4. Correlation Analysis

The clinical significance of blood basophil counts in the study is demonstrated in Table 3. For EOA patients, the percentage of blood basophils was positively correlated with the percentage of blood eosinophils (*r* = 0.549, *p*=0.002) and the absolute value of blood eosinophils (*r* = 0.496, *p* < 0.001). Although blood basophils were increased in LOA (*p* < 0.05) compared to COPD and healthy controls, this was not correlated with any clinical parameter in the LOA group (Table 2).

## 4. Discussion

This was a single-center, population-based, cross-sectional study to describe the clinical characteristics in patients with LOA, with an emphasis on assessing the natural disease course without pharmacologic interventions. The main findings of the study include: (1) Atopy was less common in LOA compared to EOA, but the antigen sensitization profiles were different. Sensitization to mites was most frequent in EOA patients, followed by LOA and COPD. Subjects with LOA were significantly more likely than those with EOA to exhibit sensitization to mold. (2) Spirometry values were lower in patients with LOA compared to EOA at their initial presentation. (3) Blood eosinophils were a typical feature in both the LOA and EOA groups. Number of blood basophils were also increased and correlated with eosinophils in EOA but not with LOA. There were no differences in blood neutrophils among subjects with LOA or EOA and healthy controls.

Traditionally, LOA was considered to have less of an allergic component, as an incomplete response to bronchodilators [24], and is more associated with obesity [25] and neutrophilic inflammation [10, 26]. However, LOA patients enrolled in previous investigations were taking ICS, OCS, or leukotriene receptor blocker during the study period, which would interfere with the analyses of disease features. Elderly females appear at a greater risk of asthma than elderly males [1], especially if they are obese [15, 27]. In this study, there were a higher number of females in the LOA group with an increased BMI compared to the EOA group. This corroborates with the report by Tomita et al. that female obesity is a risk factor for LOA [28]. Interestingly, BMI values in females with COPD and LOA were similar (Table 1). Further studies are needed to confirm the results and to identify the mechanism.

Our findings showed a major difference in the atopic sensitization between LOA and EOA. The Epidemiology and Natural History of Asthma (TENOR) study found that subjects with LOA had less allergic rhinitis and fewer patients with sIgE to at least one allergen [29], which is in accordance with the current study. However, antigen sensitization differed between the LOA and EOA groups. Sensitization to mites was most common in the EOA group, followed by LOA and COPD. Sensitization to mold was significantly more likely in LOA compared to EOA. Furthermore, *Aspergillus fumigatus* sensitivity was increased in LOA compared to EOA. This suggests that sensitization to mold in patients with LOA may be related to their living and work environment [30], and epigenetic changes may also contribute to different phenotypes of asthma [31, 32].

Compared to younger cohorts, subjects with LOA have more severe asthma and higher rates of AHR. In our study, LOA patients were more likely to be categorized as moderate (52.3% vs. 46.7%%) or severe (25.2% vs. 14.4%) compared to those with EOA. Spirometry values were also significantly lower in patients with LOA compared to EOA at their initial presentation, which is similar to a previous report [33]. However, post-bronchodilator FEV_1_values in bronchodilator reversibility testing were higher in both LOA and EOA groups compared to COPD. A reasonable explanation for this is age-related physiological changes that may act synergistically with asthma to worsen airflow obstruction, including less elastic recoil, increased airway remodeling in smaller airways, increased thickness of the central airway wall, and increased inflammation [12].

Eosinophilic inflammation is a typical feature of asthma. Many studies reported that levels of blood eosinophils were similar to those of airway eosinophils in asthma [34, 35]. This is similar to our finding that blood eosinophils were increased in both LOA and EOA compared to COPD and healthy controls. Other studies reported that airway basophils were increased and correlated with airway and blood eosinophil levels, whereas airway and blood basophils were not correlated [11, 36]. Indeed, basophils are rare granulocytes in the blood and have been considered as effector cells in allergic disease [37, 38]. When we divided asthma patients into LOA and EOA cohorts, blood basophils were increased in both LOA and EOA compared to COPD and controls. Interestingly, blood basophils correlated with the percentage of blood eosinophils and the absolute value of blood eosinophils in EOA but not in LOA. The proportion or composition of inflammatory cells may contribute to pathophysiological differences between EOA and LOA. Finally, there were no differences in blood neutrophils among EOA, LOA, and healthy controls in the study, which is in accordance with a previous report that neutrophils were not associated with LOA [39]. Further studies are required to substantiate our findings. Although previous studies have tried to address the factors associated with the development of EOA and LOA, there are no prediction models for determining the risk factors for developing EOA and LOA. Our current study provided insights into the probable factors associated with EOA and LOA. The causal relationships need to be elucidated in future studies.

There are limitations to our study. Airway inflammation could not be assessed as sputum induction was not performed due to the high concentration of saline, which could exacerbate asthma symptoms in patients without medication-controlled disease [11]. LOA with overlapping COPD was also difficult to distinguish in this cross-sectional study [1]. Furthermore, the cross-sectional nature of the study may also introduce reporting bias and since some data were self-reported by the subjects, there could also be recall bias. Similarly, since our medical center is exclusive for adult patients, we have not included children and adolescents with EOA which could have contributed to inclusion bias of the study. Nevertheless, the large representative sample included in the study negates the limitations to a certain extent.

## 5. Conclusions

LOA is a distinct clinical entity from EOA. In LOA, atopy was less frequent compared to EOA and antigen sensitization differences may be related to their living and work environments. Spirometry values were lower for LOA patients at their initial presentation than EOA patients. In EOA, blood basophils and eosinophils were significantly correlated.

COPD, chronic obstructive pulmonary disease; EOA, early-onset asthma; LOA, late-onset asthma.

## Figures and Tables

**Table 1:** Patient clinical characteristics.

Characteristic	EOA	LOA	COPD
Patients (n)	90	111	106
Age (years)	28.73 ± 5.89	60.25 ± 9.85	61.58 ± 10.95
Age (range in years)	18–39	41–80	38–84
Male (%)	54 (60)	66 (59.5)	76 (71.7)
Female (%)	36 (40)	45 (40.5)	30 (28.3)
Weight (kg)	67.45 ± 13.7	65.32 ± 11.49	66.65 ± 10.56
Height (m)	1.7 ± 0.09	1.65 ± 0.08	1.67 ± 0.07
BMI (kg/m^2)^	23.26 ± 3.37	24.1 ± 3.78	23.96 ± 3.46
BMI male	24.24 ± 3.57	24.09 ± 3.68	23.76 ± 3.36
BMI female	21.78 ± 2.50	24.11 ± 4.00^∗^	24.48 ± 3.80
Smoking history (%)	5 (5.6)	38 (25)	66 (62.3)
Family history (%)	63 (70)	36 (32.1)	17 (16)
Rhinitis (%)	72 (80)	59 (52.7)	13 (12.3)
Asthma severity			
Mild	35 (38.9)	25 (22.5)	—
Moderate	42 (46.7)	58 (52.3)	—
Severe	13 (14.4)	28 (25.2)	—
GOLD			
GOLD1	—	—	42 (39.6)
GOLD2	—	—	31 (29.2)
GOLD3	—	—	26 (24.5)
GOLD4	—	—	7 (6.6)

Data are shown as mean±SD or *n* (%). BMI, body mass index; COPD, chronic obstructive pulmonary disease; EOA, early-onset asthma; GOLD, Global Initiative for Chronic Obstructive Lung Disease; LOA, late-onset asthma. ^∗^*p* < 0.05 LOA vs. EOA.

**Table 2:** Blood cells count data of participants.

Characteristic	EOA	LOA	COPD	LOA-controls	EOA-controls
	*n* = 90	*n* = 111	*n* = 106	*n* = 30	*n* = 30
White cell counts (×10^9^/L)	7.11 ± 1.84	6.97 ± 1.99	6.58 ± 1.77	6.34 ± 1.11	6.68 ± 0.9
Blood eosinophils (%)	4.54 ± 2.80∗	4.63 ± 4.39^∗^	1.64 ± 1.09	2.2 ± 1.7	1.88 ± 1.03
Blood eosinophils (×10^9^/L)	0.33 ± 0.24∗	0.33 ± 0.34^∗^	0.10 ± 0.07	0.14 ± 0.11	0.12 ± 0.06
Blood basophils (%)	0.86 ± 0.44†	0.65 ± 0.4^#^	0.5 ± 0.24	0.47 ± 0.23	0.48 ± 0.19
Blood basophils (×10^9^/L)	0.07 ± 0.04†	0.05 ± 0.04^#^	0.04 ± 0.02	0.04 ± 0.02	0.04 ± 0.02
Blood neutrophils (%)	54.51 ± 9.04	55.01 ± 16.55	59.73 ± 17.18	56.17 ± 7.28	56.38 ± 6.39
Blood neutrophils (×10^9^/L)	4.83 ± 3.94	6.13 ± 10.09	6.44 ± 11.99	3.54 ± 0.91	3.87 ± 0.88

Data are shown as mean ± SD. COPD, chronic obstructive pulmonary disease; EOA, early-onset asthma; LOA, late-onset asthma. ^∗^*p* < 0.01 EOA and LOA vs. COPD and LOA-controls and EOA-controls; ^#^*p* < 0.05 LOA vs. EOA and COPD and LOA-controls and EOA-controls; ^†^*p* < 0.01 EOA vs. COPD and LOA-controls and EOA-controls.

**Table 3:** Relationship between blood basophils (%) and clinical data.

EOA (*n* = 90)		LOA (*n* = 111)	
Blood basophils%	*r*	*p* value	*r*	*p* value
Gender	0.043	0.823	0.135	0.476
BMI	0.132	0.488	0.211	0.262
Smoking history	0.222	0.238	−0.172	0.363
Family history	−0.019	0.921	0.141	0.458
Rhinitis	−0.119	0.532	−0.116	0.541
White cell counts (^∗^10^9^/L)	−0.004	0.983	−0.194	0.313
Blood eosinophils (%)	0.549	0.002	0.317	0.088
Blood eosinophils (^∗^10^9^/L)	0.496	0.000	0.227	0.228
Blood basophils (^∗^10^9^/L)	0.937	0.000	0.800	0.000
Blood neutrophils (%)	0.036	0.849	−0.021	0.913
Blood neutrophils (^∗^10^9^/L)	−0.143	0.451	−0.281	0.140
Spirometry				
FEV_1_/FVC (%)	−0.199	0.362	−0.046	0.818
FEV_1_% pred	−0.062	0.779	0.024	0.904
FEV_1_ (L)	−0.056	0.8	0.171	0.394
FVC% pred	−0.098	0.665	0.179	0.373
FeNO (ppb)	−0.031	0.877	0.179	0.414
Allergen				
Mites	−0.084	0.659	−0.064	0.737
Cat fur	−0.14	0.461	0.173	0.361
Dog fur	−0.079	0.679	0.213	0.258
Cockroach	0.069	0.716	−0.027	0.889
Wheat flour	−0.228	0.226	−0.176	0.351
Egg	−0.084	0.061	0.173	0.361
Molds	—	—	−0.077	0.688
*Aspergillus fumigatus*	—	—	−0.077	0.688
Willow	—	—	0.173	0.361
Weeds	0.239	0.203	0.123	0.517
sIgE (kIU/L)	0.012	0.951	0.026	0.917

BMI, body mass index; EOA, early-onset asthma; FeNO, fractional exhaled nitric oxide; FEV_1_, forced expiratory volume in 1 second; FVC, forced vital capacity; LOA, late-onset asthma; pred, predicted; sIgE, specific immunoglobulin E.

## Data Availability

All data or resources used in the current study are available from the corresponding author upon reasonable request.

## Supplementary Materials

Supplementary Materials Table S1: lung function test and allergy status in the study.
