# Supplementary material for: Clinical Differences between Early- and Late-Onset Asthma: A Population-Based Cross-Sectional Study
Source: Can Respir J. 2021 Jan 12;2021:8886520. doi: 10.1155/2021/8886520 (PMC7864752; doi:10.1155/2021/8886520)
Supplement: Supplementary Materials — Table S1: lung function test and allergy status in the study. [file 8886520.f1.pdf]

Table S1 Lung function test and allergy status in the study

Characteristic	LOA	LSA(EOA)	COPD
	n=111	n=90	n=106
Spirometry			
FEV $_1$/FVC (%)	63.68±11.53	79.77±7.61 #	63.41±7.84
FEV $_1$/FVC%pred	80.82±14.3	93.91±7.95 #	78.48±9.73
FEV $_1$%pred	68.56±23.9	96.67±13.84 #	70.05±19.11
FEV $_1$(L)	1.8±0.77	3.43±0.713 #	2.01±0.71
FVC %pred	81.74±22.94	105.46±14.1 #	86.98±21.24
Bronchodilator reversibility			
FEV $_1$% change	12.44±5.48	10.46±12.31	2.78±3.76*
FEV $_1$(L) change	0.27±0.17	0.22±0.15	0.01±0.24*
FeNO	55.81±47.15	71.03±62.74	17.38±6.46*
Allergen			
Mites	48 (42.9) †	74 (82.2) ‡	5 (4.7)
Cat	11 (9.8)	21 (23.3) ‡	0
Dog	23 (20.5)	16 (17.8)	5 (4.7)
Cockroach	8 (7.1)	18 (20)	2 (1.9)
Wheat flour	11 (9.8)	3 (3.3)	0
Egg	4 (3.6)	8 (8.9)	0
Molds	16 (14.3) †	1 (1.1)	2 (1.9)
Aspergillus fumigatus	15 (13.4)	1(1.1)	1 (0.9)
Willow	7 (6.2)	0	0
Weeds	6 (5.4)	1 (1.1)	2 (1.9)
sIgE (KIU/L)	219.17±331.7	241.03±334.7	50.16±64.21*

Data are shown as mean ±(SD) or No. (%), LOA, late-onset asthma; LSA, long-standing asthma;

#p<0.01 LOA & COPD *vs.* LSA; * p<0.01 LOA & LSA *vs.* COPD; †p<0.05 LOA *vs.*LSA; ‡p<0.01 LSA *vs.* COPD.
